# Effect of lumbar spinal stenosis on bone mineral density in osteoporosis patients treated with ibandronate

**DOI:** 10.1186/s12891-021-04273-x

**Published:** 2021-05-04

**Authors:** Hyung-Youl Park, Ji-Yoon Ha, Ki-Won Kim, In-Hwa Baek, Soo-Bin Park, Jun-Seok Lee

**Affiliations:** 1 Department of Orthopedic Surgery, Eunpyeong St. Mary’s Hospital, College of Medicine, the Catholic University of Korea, 1021, Tongil-Ro, Eunpyeong-gu, 03312 Seoul, Republic of Korea; 2 Department of Orthopedic Surgery, Yeouido St. Mary’s Hospital, College of Medicine, the Catholic University of Korea, Seoul, Korea

**Keywords:** Osteoporosis, Lumbar spinal stenosis, Ibandronate, Bone mineral density

## Abstract

**Background:**

Lumbar spinal stenosis (LSS) can cause various neurological symptoms and reduce the daily activity of patients. Many studies have shown that free physical activities and exercise can improve bone mineral density (BMD) in patients with osteoporosis. However, the effect of LSS on BMD has not been reported. The purpose of this study was to investigate the effects of LSS on BMD in patients treated with ibandronate for newly diagnosed osteoporosis.

**Methods:**

Group 1 included 83 patients treated for osteoporosis alone, and group 2 included 76 patients treated for both osteoporosis and symptomatic LSS. We confirmed four BMD values presented as T-score at initial, and 1-, 2-, and 3-year follow-ups. Mean BMD and annual changes of BMD for three years were compared between the two groups. Correlations between initial BMD and total change of BMD, and related factors for continuous BMD improvement for three years were also evaluated.

**Results:**

Mean annual BMDs were significantly higher in group 1 compared than in group 2 (-3.39 vs. -3.58 at 1-year; -3.27 vs. -3.49 at 2-year; -3.13 vs. -3.45 at 3-year; all *p* < 0.05). Annual change of BMD at 1-year follow-up (0.32 vs. 0.21, *p* = 0.036) and total change of BMD for three years (0.57 vs. 0.35, *p* = 0.002) were significantly higher in group 1. Group 1 had a strong negative correlation (*r* = -0.511, *P* = 0.000) between initial BMD and total change of BMD, whereas group 2 showed a weak negative correlation (*r* = -0.247, *p* = 0.032). In multivariate analysis, symptomatic LSS was the only independent risk factor for continuous BMD improvement (Odds ratio = 0.316, *p* = 0.001).

**Conclusions:**

Symptomatic LSS may interfere with BMD improvement in the treatment of osteoporosis with ibandronate. Active treatment for LSS with more potent treatment for osteoporosis should be taken to increase BMD for patients with osteoporosis and LSS.

## Background

Osteoporosis is a systemic skeletal disorder in which bones become weak and vulnerable [1, 2]. Bisphosphonates are the potent bone resorption inhibitors. They include alendronate, risedronate, zoledronic acid, and ibandronate, which are widely used for the treatment of osteoporosis [3]. The action mechanism of bisphosphonate is that it can bind to an enzyme which acts in the pathway of osteoclast to inhibit the synthesis of cholesterol and decrease the formation of the proteins necessary for osteoclast function and survival, inducing osteoclast cell death [4]. Particularly, ibandronate can significantly reduce the risk of new vertebral fractures and increase bone mineral density (BMD) at the lumbar spine in postmenopausal women [5].

Lumbar spinal stenosis (LSS) is commonly associated with degenerative changes in which the spinal canal, lateral recess, and intervertebral foramen are narrowed and compress the cauda equina or nerve roots [6]. It can cause various neurological symptoms, such as lower back pain, intermittent claudication, and gait disturbance [7]. These neurological symptoms are known to reduce the daily activities of patients with LSS more than those of patients without LSS [8, 9]. Many studies have shown that physical activity and exercise can improve BMD in patients with osteoporosis [10–12].

The prevalence of osteoporosis is continuing to escalate with the increasing elderly population [1]. In the United States, the estimated prevalence was 10.3 % (10.2 million) among adults aged 50 years or older in 2010 [13]. Lee et al. [14] have reported that 22.6 % of postmenopausal patients with symptomatic LSS have osteoporosis, and 41.5 % require treatment for osteoporosis in a cross-sectional study. As the prevalence of osteoporosis increases, studies on osteoporosis have also increased. However, to the best of our knowledge, the effect of LSS on BMD has not been reported. We hypothesized that LSS would have negative effect on BMD, because LSS causes neurological symptoms and eventually reduces the patient’s physical activity. We aimed to evaluate the effect of LSS on BMD in patients undergoing osteoporosis treatment with ibandronate.

## Materials and methods

### Study population

From January 2004 to December 2017, we retrospectively reviewed 398 patients in a single institution who had been initially diagnosed with postmenopausal osteoporosis and who had started taking osteoporosis medications. All patients included in this study visited the outpatient spine clinic. We divided the patients into two groups according to whether patients were being treated for symptomatic LSS at the time of initial diagnosis for osteoporosis. Patients treated for osteoporosis alone visited outpatient clinic for regular follow-up. On the other hand, patients treated for both osteoporosis and LSS visited the outpatient clinic for LSS symptoms and started taking medications for newly diagnosed osteoporosis.

Inclusion criteria were: (1) undergoing BMD evaluation every year for three years after the initial BMD test in patients with newly diagnosed osteoporosis (a total of four BMD evaluations), and (2) 150 mg once-monthly oral ibandronate treatment for three years. All patients received calcium (600 mg/day) and vitamin D (400 international units/day) as an adjunctive therapy.

Exclusion criteria were: (1) taking steroid hormones, (2) surgery or fracture of spine or lower extremities, (3) malignant tumor or medical conditions that reduced daily activities, (4) rheumatoid arthritis, (5) metabolic bone disease, and (6) gait disorders due to causes other than LSS. Finally, 159 patients were included in this study.

Clinical parameters, including age, sex, body mass index (BMI), underlying diseases such as hypertension and diabetes, and BMD, were assessed by means of patients’ electronic medical records. This study was approved by the Institutional Review Board (IRB) in accordance with the Declaration of Helsinki (approval number. PC18RESE0034). The informed consents were waived by IRB (The Catholic University of Korea, Eunpyeong St. Mary’s Hospital) because of the retrospective study design.

### Diagnosis and treatment of symptomatic LSS

Symptomatic LSS were confirmed as obvious central stenosis on magnetic resonance imaging (MRI) and clinically reated neurological symptoms. According to MRI-based classification system for central stenosis by Lee et al. [15], patients with grade 2 or 3 central stenosis were included. Patients with concomitant foraminal stenosis or lateral recess stenosis, combined with central stenosis, were also included [16, 17]. Clinically related neurological symptoms were as following: (1) pain, weakness or numbness in the legs, calves or buttocks when standing or walking, (2) neurological claudication; cramping in the calves with walking, requiring frequent short rests to walk a distance, and (3) symptoms that improve when sitting or bending forward [6].

**Fig. 1:**
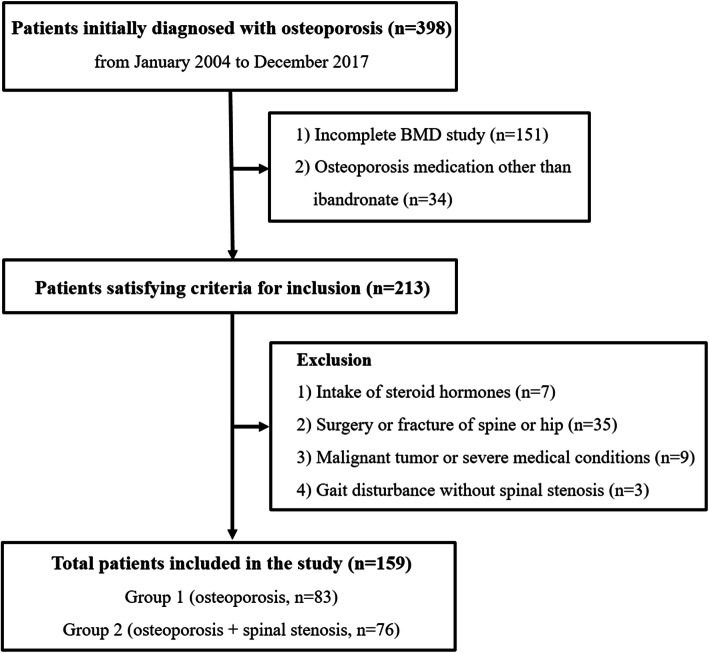
Flow chart showing the inclusion of patients in this study

Patients who treated for osteoporosis alone without LSS symptoms were included as group 1 (osteoporosis group), while group 2 included patients treated for both osteoporosis and symptomatic LSS (osteoporosis + spinal stenosis group) (Fig. 1). All patients with LSS were treated conservatively with medications and physical therapy. Medications included nonsteroidal anti-inflammatory drugs for pain control and pregabalin, gabapentin or opioids if tolerated. Patients who underwent surgery for worsening of stenosis during the follow-up period were excluded from this study.

### Measurement of BMD

Osteoporosis was confirmed by dual-energy X-ray absorptiometry (Lunar Prodigy; GE Healthcare Bio-Sciences Corp., Piscataway, NJ, USA) for all patients. A BMD T-score of -2.5 or less in the total lumbar spine or femur (total or neck) was defined as osteoporosis [18].

We confirmed a total of four BMD values presented as T-score, initial and at 1-, 2-, and 3-year follow-ups, in each patient at the total lumbar spine or femur (total or neck), where the initial BMD was assessed. We obtained annual changes of BMD in each group by calculating the difference in mean BMDs for two consecutive years (annual change of BMD = BMD in index year – BMD in the previous year).

### Evaluation of BMD improvement and related factors

We assessed a total change of BMD for three years by calculating the difference between initial mean BMD and mean BMD at three years later (a total change of BMD = BMD on 3-year follow-up – initial BMD). To investigate the improvement of osteoporosis according to the initial BMD in each group, we assessed correlations between initial BMD and total change of BMD in both groups. Finally, univariate and multivariate analyses were done to identify the related factors for continuous improvement of BMD for three years.

### Statistical analyses

We compared means of continuous variables using a paired *t*-test within each group and unpaired Student’s *t*-test between the groups. The correlation between initial BMD and total change of BMD was analyzed by the Pearson correlation coefficient, and simple linear regression was simultaneously conducted. Variable with *p* value < 0.1 on univariate analysis was included in multivariate analysis using logistic regression test. All statistical analyses were done using the SPSS software (IBM SPSS Statistics, Version 24.0, IBM Corp., Armonk, NY, USA). Values for *p* < 0.05 were considered significant.

## Results

Table 1 shows patient characteristics between the two groups. The number of patients was 83 in group 1, and 76 in group 2. There were no significant differences in mean age, BMI, underlying diseases, and initial BMD between the two groups.

**Table 1:** Patient characteristics between the two groups

	Group 1 (osteoporosis)	Group 2 (osteoporosis + LSS)	*P*-value
Patients number	83	76	
Female : male	83 : 0	76 : 0	1.000
Age (years)	73.7 ± 9.6	74.8 ± 8.9	0.492
Height (cm)	154.2 ± 8.0	152.1 ± 4.8	0.284
Weight (kg)	56.3 ± 9.8	53.6 ± 8.5	0.352
BMI (kg/m^2^)	23.6 ± 3.3	23.1 ± 3.0	0.609
Underlying diseases			
Hypertension	30 (36.1 %)	29 (38.2 %)	0.793
Diabetes	18 (21.7 %)	16 (21.1 %)	0.922
Initial BMD (T-score)	-3.71 ± 0.49	-3.80 ± 0.77	0.388

*LSS* lumbar spinal stenosis, *BMI* body mass index, *BMD* bone mineral density

### Comparison within the group

**Fig. 2:**
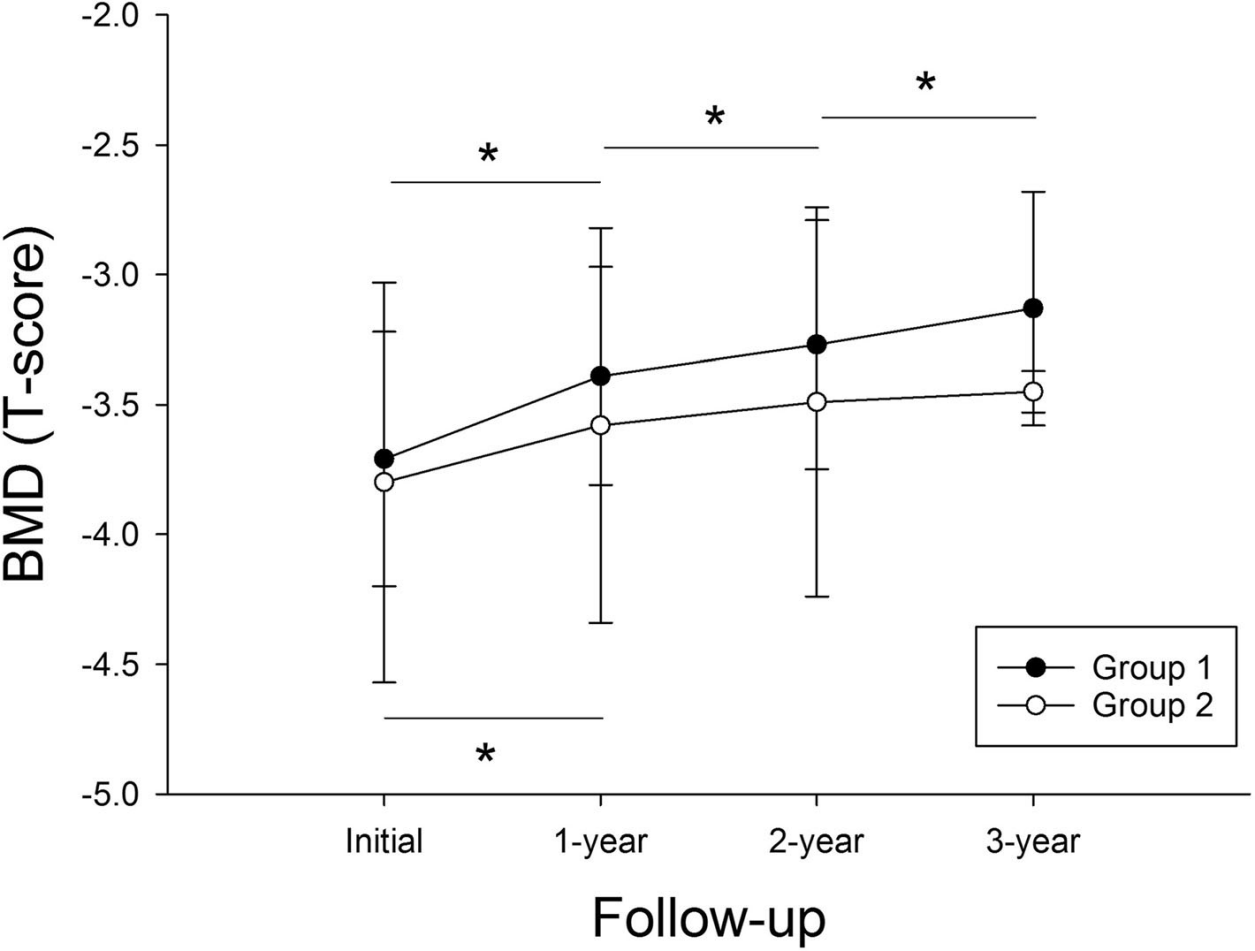
Annual BMDs in the two groups. * indicates *p* < 0.05

In group 1, mean BMDs improved significantly every year during the three-year follow-up (-3.71 ± 0.49 vs. -3.39 ± 0.42 vs. -3.27 ± 0.48 vs. -3.13 ± 0.45, all *p <* 0.001) (Fig. 2). In group 2, mean BMD at 1-year follow-up improved significantly compared to the initial BMD (-3.80 ± 0.77 vs. -3.58 ± 0.76, *p* < 0.001). However, mean BMDs at 2- and 3-year follow-ups did not improve significantly compared to the previous year (-3.58 ± 0.76 vs. -3.49 ± 0.75, *p* = 0.064; -3.49 ± 0.75 vs. -3.45 ± 0.80, *p* = 0.424) (Fig. 2).

### Comparison between the two groups

Table 2 shows the mean BMDs and annual changes of BMDs during follow-up. Mean BMDs at annual follow-up for three years were significantly higher in group 1 than in group 2. The annual change of BMD at the 1-year follow-up was significantly higher in group 1 than in group 2. However, there were no differences in the annual change of BMD at the 2-year and 3-year follow-ups between the two groups. A total change of BMD for three years was also significantly higher in group 1 than in group 2.

**Table 2:** BMD, annual change of BMD, and a total change of BMD in the two groups

	Group 1 (osteoporosis)	Group 2 (osteoporosis + LSS)	*P*-value
BMD			
Initial	-3.71 ± 0.49	-3.80 ± 0.77	0.388
at 1-year F/U	-3.39 ± 0.42	-3.58 ± 0.76	**0.045**
at 2-year F/U	-3.27 ± 0.48	-3.49 ± 0.75	**0.034**
at 3-year F/U	-3.13 ± 0.45	-3.45 ± 0.80	**0.003**
Annual change of BMD			
at 1-year F/U	0.32 ± 0.29	0.21 ± 0.34	**0.036**
at 2-year F/U	0.12 ± 0.28	0.09 ± 0.44	0.702
at 3-year F/U	0.14 ± 0.25	0.04 ± 0.41	0.069
Total change of BMD	0.57 ± 0.41	0.35 ± 0.50	**0.002**

*LSS* lumbar spinal stenosis, *BMD* bone mineral density, *F/U* follow-up

### Correlation between initial BMD and total change of BMD in both groups

**Fig. 3:**
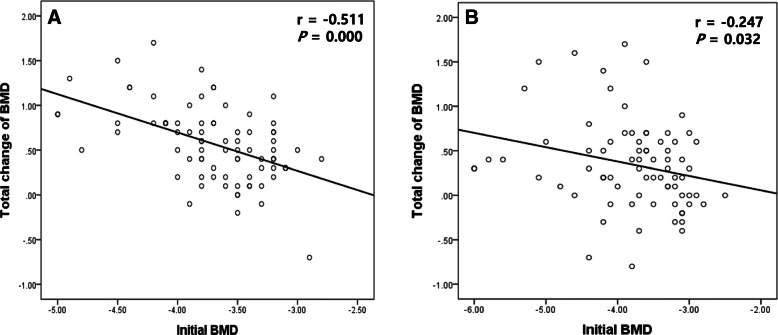
Correlations between initial BMD and total change of BMD in (**a**) group 1 and (**b**) group 2

Group 1 showed a strong negative correlation between initial BMD and total change of BMD (*r* = -0.511, *p* = 0.000; Fig. 3a). Group 2 showed a weak negative correlation between initial BMD and total change of BMD (*r* = -0.247, *p* = 0.032; Fig. 3b).

### Univariate and multivariate analyses for continuous BMD improvement

Continuous improvement of BMD for three years was observed in 71 patients, while 88 patients had non-continuous improvement. Age, BMI, underlying diseases, and initial BMD were similar between two groups (Table 3). The prevalence of symptomatic LSS was significantly lower in the continuous improvement group (32.4 % vs. 60.2 %, *p* = 0.000). In multivariate analysis, the only independent factor affecting BMD improvement was the symptomatic LSS (odds ratio = 0.316, [95 % confidence interval 0.164–0.609]; *p* = 0.001).

**Table 3:** Univariate and multivariate analyses for continuous improvement of BMD for three years

**Univariate analysis**	**Continuous improvement**	**Non-continuous improvement**	***P***-value
Patients number	71	88	
Age (years)	75.5 ± 10.1	73.2 ± 8.5	0.125
BMI (kg/m^2^)	22.6 ± 3.5	23.5 ± 2.9	0.382
Hypertension	27 (38.0 %)	32 (36.4 %)	0.829
Diabetes	14 (19.7 %)	20 (22.7 %)	0.645
Initial BMD (T-score)	-3.76 ± 0.72	-3.74 ± 0.52	0.869
Symptomatic LSS	23 (32.4 %)	53 (60.2 %)	**0.000**
**Multivariate analysis**	**Odds ratio**	**95 % Confidence interval**	***P*-value**
Symptomatic LSS	0.316	0.164–0.609	**0.001**

*BMD* bone mineral density, *BMI* body mass index, *LSS* lumbar spinal stenosis

## Discussion

We found that ibandronate increased BMDs in patients newly diagnosed with osteoporosis. In both groups, the mean BMD at the 3-year follow-up improved significantly compared to the initial value (-3.71 vs. -3.13 for group 1; -3.80 vs. -3.45 for group 2, all *p <* 0.001). This result was consistent with a previous report showing that BMDs at the lumbar spine and hip increased significantly (3.1 and 1.8 %, respectively; *p <* 0.0001 vs. placebo) with daily 2.5-mg ibandronate after 24 months [19].

In patients with osteoporosis alone, the annual BMD increased significantly over the previous BMD for three years, which suggests that the therapeutic effect of ibandronate is significant every year in patients with osteoporosis alone. In patients with osteoporosis and LSS, the annual BMD increased significantly from the initial value only during the first year but did not show a significant increase after that. Moreover, annual change at 1st year follow-up (0.32 vs. 0.21, *p* = 0.036) and total change for three years (0.57 vs. 0.35, *p* = 0.002) were significantly greater in patients with osteoporosis alone than in patients with both osteoporosis and LSS.

LSS causes neurologic claudication and reduces the strength of the lower limb, which decreases physical activity [10]. Walking difficulty due to claudication or physical inactivity can be associated with decreased BMD [20]. A previous study reported a relationship between physical inactivity and decreased BMD in patients with vascular claudication originating from peripheral arterial disease [20]. Physical activities in seniors can induce the maintenance of BMD or increase BMD by means of physical load [11, 21, 22]. Lee et al. [23] have also reported that increased physical activity and regular walking exercise could prevent osteoporosis in a study of older women aged 65 years and over. In the present study, the direct comparison of annual BMDs between the two groups over three years showed that the annual BMDs in patients with osteoporosis and LSS were significantly lower than in patients with osteoporosis alone, which suggests that LSS may interfere with the improvement of BMD in the treatment of osteoporosis.

The correlation analysis between initial BMD and total change of BMD means the relationship between the initial osteoporosis and osteoporosis improvement after treatment, which suggests the different therapeutic efficacies of ibandronate in treatment of osteoporosis. Strong negative correlation in patients with osteoporosis alone suggested the better efficacy of ibandronate than in patients with both osteoporosis and LSS showing the weak negative correlation. Moreover, univariate and multivariate analyses revealed that symptomatic LSS was the significant risk factor for continuous improvement of BMD in patients undergoing osteoporosis treatment with ibandronate.

In patients with LSS, a decrease in the therapeutic effect of ibandronate may also be associated with deterioration of bone metabolism [24]. Lee et al. [14] reported that 55.6 % of patients with LSS had hypovitaminosis D, which reduced the effectiveness of osteoporosis treatment. Kim et al. [25] reported that limited physical activity in symptomatic LSS patients resulted in high bone turnover rates, including bone formation and bone resorption markers. Moreover, in a subsequent study, they reported that decompression surgery for symptomatic LSS patients had a positive effect on bone metabolism by reducing the increased bone resorption rate [26]. These reports show that improved walking ability and physical activity resulting from the active treatment of LSS can help improve the effectiveness of osteoporosis treatment. Therefore, active treatment for LSS to alleviate neurological symptoms, combined with more potent treatment for osteoporosis should be taken to increase BMD for patients with osteoporosis and LSS.

This study has some limitations. First, we did not make an objective measure that daily activity was lower in patients with osteoporosis and LSS than in patients with osteoporosis alone. Previous studies have reported that daily activity decreases in patients with LSS, so we were able to conduct this study based on the earlier results [8–10]. Further research using objective tools, such as questionnaires on daily activities, is needed. Second, we could not include bone turnover markers in this study. Because it was retrospective, test values were not present for some patients. Third, this study included only patients treated with ibandronate. Thus, the results of this study may not apply to patients treated with other types of osteoporosis drugs. Additional prospective trials are needed to validate our findings and extend these results to different kinds of osteoporosis drugs. Finally, clinical outcomes of osteoporosis treatment, such as osteoporotic fractures, were not evaluated during the follow-up, because we focused only on whether LSS affects BMD in the treatment of osteoporosis. Despite these limitations, our study has the strength that it is the first case series to evaluate the effect of LSS on BMD in the treatment of osteoporosis patients.

## Conclusions

We have demonstrated that BMD increased less in patients with osteoporosis and LSS than in patients with osteoporosis alone during osteoporosis treatment with ibandronate. Symptomatic LSS was also the independent risk factor for continuous improvement of BMD. This result supported that symptomatic LSS may interfere with BMD improvement in the treatment of osteoporosis patients. Therefore, active treatment for LSS to increase the physical activities, combined with more potent treatment for osteoporosis should be taken to increase BMD for patients with osteoporosis and LSS.

## Data Availability

The datasets generated and/or analyzed during the current study are available from the corresponding author on reasonable request.

## Abbreviations

BMD: Bone mineral density
LSS: Lumbar spinal stenosis
BMI: Body mass index
IRB: Institutional Review Board
MRI: Magnetic resonance imaging
